# New Evidence in the Pathogenesis of Celiac Disease and Type 1 Diabetes Mellitus: A Systematic Review

**DOI:** 10.7759/cureus.16721

**Published:** 2021-07-29

**Authors:** Jose Prieto, Karan B Singh, Maduka C Nnadozie, Muhammad Abdal, Niki Shrestha, Rose Anne M Abe, Anum Masroor, Arseni Khorochkov, Lubna Mohammed

**Affiliations:** 1 Internal Medicine, California Institute of Behavioral Neurosciences & Psychology, Fairfield, USA; 2 Research, California Institute of Behavioral Neurosciences & Psychology, Fairfield, USA; 3 Emergency Medicine, California Institute of Behavioral Neurosciences & Psychology, Fairfield, USA; 4 Psychiatry, California Institute of Behavioral Neurosciences & Psychology, Fairfield, USA; 5 Psychiatry, Psychiatric Care Associates, Englewood, USA; 6 Medicine, Khyber Medical College, Peshawar, PAK

**Keywords:** celiac disease, type 1 diabetes mellitus (t1dm), human leukocyte antigen (hla), microbiota, autoimmune, immune profile

## Abstract

Celiac disease (CD) and type 1 diabetes mellitus (T1DM) are autoimmune diseases that coexist frequently. These illnesses share a common genetic background. This study aims to review the different pathophysiologic mechanisms that have been studied about the coexistence of CD and T1DM, to contrast them, and to summarize their specific role in these autoimmune diseases. We conducted a systematic review following the Preferred Reporting Items for Systematic reviews and Meta-Analyses (PRISMA) checklist and used the Medical Subject Headings (MeSH) search strategy to obtain relevant articles. We found 585 papers which were reduced to 355 after removing duplicates. Later, the filters and inclusion/exclusion criteria were applied which ended the search with 78 articles. Finally, we reviewed the articles that contained information about the pathogenesis of CD and T1DM, their coexistence, and how the pathogenesis impacts clinical outcomes. The reviewed studies strongly conclude that the presence of human leukocyte antigen (HLA) genes DQ2 and DQ8 are high-risk for developing the coexistence of CD and T1DM. We found that killer immunoglobulin-like receptor (KIR) genes, enterovirus infection in gut cells, and gut microbiota dysbiosis with the predominance of *Bacteroides *spp. also play a role in the pathogenesis and development of symptoms of CD in patients with the previous diagnosis of T1DM. CD4+ and CD8+ cell levels vary among patients and studies, consequently, more study on this topic is needed.

## Introduction and background

Celiac disease (CD) and type 1 diabetes mellitus (T1DM) are autoimmune diseases that affect approximately 1% and 0.5% of the general population worldwide, respectively [1]. They coexist in around 4-5% of the cases with T1DM occurring usually some years before CD [2]. CD is a disorder characterized by a hypersensitivity to gluten, a protein found in wheat, rye, barley, and others. When gluten is ingested, it causes a variety of gastrointestinal symptoms, but the most concerning is the malabsorptive syndrome produced principally by tissue transglutaminase antibodies (tTG), an IgA-mediated response [3]. Otherwise, T1DM is a classic autoimmune disorder in which there occurs an IgG-mediated pancreatic B-cell destruction by autoantibodies (glutamic acid decarboxylase antibodies - GAD) and later insulin deficiency with rising blood glucose [4].

The importance of this coexistence lies in that the patients have to deal with the symptomatology of both diseases. Usually, first, the polyuria; polydipsia; polyphagia; weight loss, all classic symptoms of diabetes, and then, the unknown development of CD and all the variety of symptoms of malabsorption and its complications. Also, the patients and their families have to manage and control their treatments with insulin dosing and a gluten-free diet, which sometimes are very troublesome [5].

It is well known that these illnesses share a common genetic background that predisposes individuals to develop them. The presence of human leukocyte antigen (HLA) type II alleles: HLA-DQ2 and HLA-DQ8, confer high risk to develop these diseases, this has been demonstrated with their presence in 98% of patients with CD and 95% of patients with T1DM [6]. However, there is rising evidence suggesting that there are some other genetic, immunological, environmental, viral exposure, and dietary factors that may contribute some importance to the pathogenesis and coexistence of these diseases in the population [7]. Additionally, studies suggest that low expression or absence of regulatory T cells, helper T cells, certain cytokines, chemokines, and other inflammatory mediators, puts individuals at risk of developing CD and T1DM [8, 9].

Even though the recent discoveries about the different pathogenic and pathophysiologic mechanisms of CD and T1DM are done in low sample studies and few places in the world, they are completely reproducible to the general population and reliable in statistic terms. That is why the research on this topic is increasing and new mechanisms are being revealed to better understand these diseases.

We aim to review the different pathophysiologic mechanisms that have been studied about the coexistence of CD and T1DM, to contrast them and summarize their specific roles in these autoimmune diseases.

Methods

We conducted a systematic review following the Preferred Reporting Items for Systematic reviews and Meta-Analyses (PRISMA) checklist. We searched articles published on PubMed until May 3, 2021. We applied the Medical Subject Headings (MeSH) search strategy to obtain related articles, and the following keywords were included: 

· Celiac disease

· Type 1 Diabetes Mellitus

· Celiac disease and Type 1 Diabetes Mellitus

Table 1 summarizes the strategy we used for MeSH terms.

**Table 1:** MeSH keywords MeSH: Medical Subject Headings

Keyword	Database	Number of results
Type 1 diabetes mellitus	PubMed	10046
Celiac disease	PubMed	1766
Celiac disease and type I diabetes mellitus	PubMed	585

Results

After using the MeSH search strategy to search for interesting articles, we found 585 papers which were then reduced to 355 after removing the duplicates. Later, the filters and inclusion/exclusion criteria were applied which ended the search with 78 articles. The titles of 78 articles were screened to identify possible relevant articles for this research. Once we finished identifying relevant papers, we screened their titles again, including the abstracts, and full text of pertinent studies.

**Figure 1:**
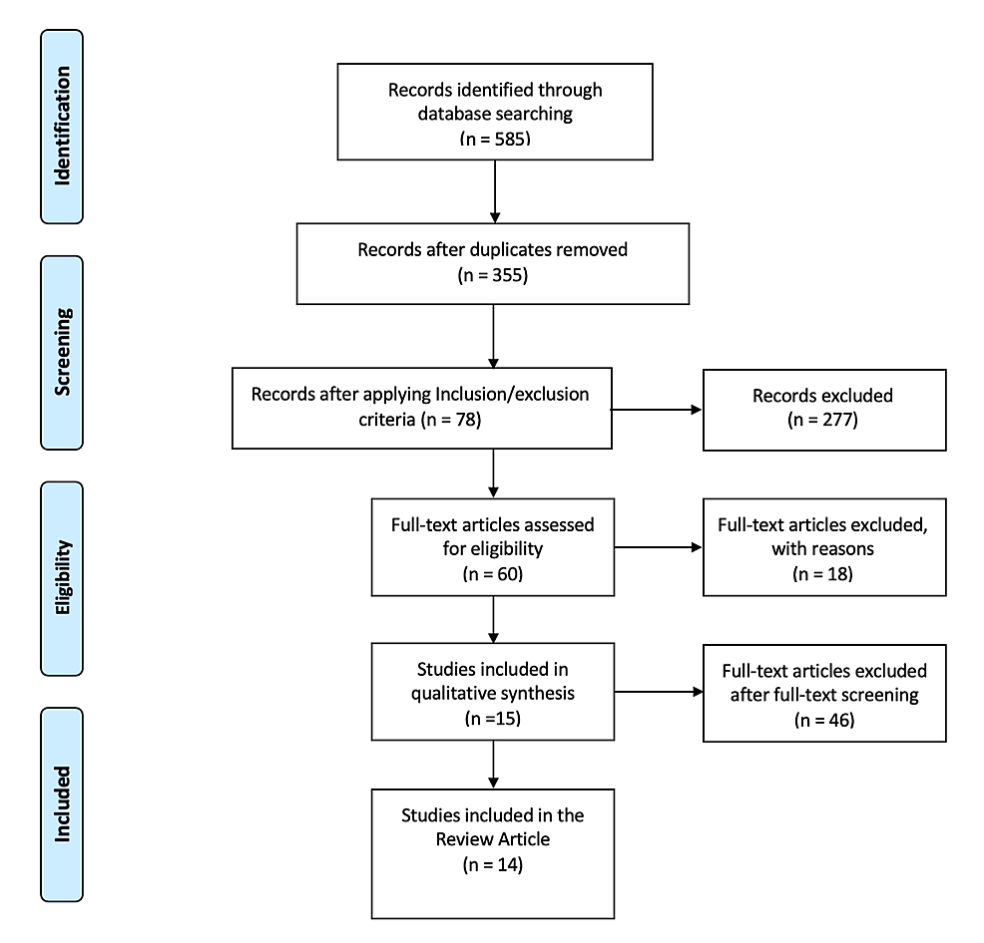
PRISMA flowchart PRISMA: preferred reporting items for systematic reviews and meta-analyses

It is important to mention that two of the authors separately reviewed the quality of the studied papers by applying different quality assessment tools, depending on the type of article, including “Newcastle-Ottawa tool” for non-randomized controlled trials and observational studies, “PRISMA checklist” for systematic reviews, and “Scale for the Assessment of Narrative Review Articles (SANRA) checklist” for literature reviews. After this quality assessment, one article was excluded leaving 14 papers to review. The entire review was done scientifically and within ethical boundaries. All these processes are summarized in Figure 1.

Inclusion criteria

We have included articles written in English in the last 10 years, done in humans. We focused on the main purpose of this research which is similarities in the pathogenesis of CD and T1DM in patients of any age and sex. All types of studies were included. All the articles included were peer-reviewed.

Exclusion criteria

Articles written in any language other than English, research done in animal models, grey literature, and published before May 3, 2011 were excluded.

## Review

Discussion

During recent years, multiple studies have demonstrated the existing relation between T1DM and CD. Various mechanisms and factors have been studied and included in these discussions, which we evaluate in this systematic review. The most established and accepted conclusion is that both diseases share a common genetic background, which is principally determined by HLA genes DQ2 and DQ8 [10].

Genetics and immunology

The importance of the association between these two diseases can be reflected in that in some countries the genotyping of HLA high-risk genes is mandatory if a patient is diagnosed with T1DM. While in other countries the coexistence of CD with T1DM has been so well established that when a patient is diagnosed with T1DM, it is not necessary to do more tests, given the high positive predictive values of these tests [11]. These studies have also shown that it is far more common for patients with T1DM to develop CD later in life than the opposite way [12]. Moreover, a big proportion of asymptomatic patients with a baseline diagnosis of T1DM also tests positive for anti-tissue transglutaminase antibodies, and its levels correlate with the degree of villous atrophy [13].

Given the importance of the association of these two diseases, some studies have been conducted to inquire into the mechanisms of this correlation and to better comprehend their behaviors. Likewise, it would help in the development of new ways to treat these sicknesses and improve the quality of life of the patients. In Table 2, we summarize the reviewed studies with the different mechanisms proposed to play a role in the correlation of T1DM and CD.

**Table 2:** Genetic and immunologic mechanisms in the correlation of type 1 diabetes and celiac disease CD: celiac disease; HLA: human leukocyte antigen; KIR: killer immunoglobulin-like receptor; T1DM: type 1 diabetes mellitus

Study	Author	Year	Type of study	Purpose of the study	Results	Conclusion
1	Siddiqui et al. [3]	2021	Cross-sectional	Evaluate the use of HLA typing for diagnosis of CD	31.4% of patients with T1DM tested positive for CD allele DQ2, 25% for allele DQ8, and 34% for both	The HLA testing can aid in the diagnosis of CD in patients without previously known disease
2	Farina et al. [14]	2019	Cross-sectional	Measure the expression of high-risk genes HLA-DQ2.5 and DQ8 and their importance in the activation of CD4 lymphocytes	Elevated expression of high-risk alleles encoding for genes: DQA1*05 and DQB1*02 coding for HLA-DQ2.5. DQA1*03 and DQB1*03 coding for HLA-DQ8	Elevated expression of high-risk alleles confers an increased risk for developing autoimmunity
3	Bhadada et al. [5]	2017	Cross-sectional	Extrapolate the biochemical, hormonal, and clinical presentations in CD vs CD+T1DM patients	CD was diagnosed in a mean of 48.8±43.4 months after T1DM diagnosis	CD is diagnosed earlier in patients with T1DM compared with patients without T1DM
4	Hagopian et al. [17]	2017	Cohort	Find out if T1DM trigger CD and if their coexistence occurs by inheritance of the same genes	The study demonstrates a 32% greater prevalence of tissue transglutaminase antibodies in patients with islet autoantibodies	T1DM and CD coexistence occurs more than expected
5	Mitchell et al. [15]	2016	Cross-sectional	Demonstrate if HLA-DQ testing for CD screening in T1DM patients is cost-effective	CD was diagnosed in 6.9% of patients studied. 94% of patients carried HLA-DQ2 and/or HLA-DQ8 which are high-risk alleles	It is not cost-effective to test all patients with T1DM for HLA-DQ genotyping due to the high rate of positive results and co-occurrence of these diseases
6	Gutierrez-Achury et al. [1]	2015	Case-control	Compare the genetic variances among patients with CD and T1DM, and patients with just one of the two diseases using genome-wide association studies (GWAS)	Eight risk alleles among 60 were present in patients with both diseases; HLA-DQ2.5 was significantly associated with double autoimmunity	Genetic risk for double autoimmunity is increased with HLA class II
7	Akar et al. [17]	2015	Cohort	Demonstrate the role of KIR genes, KIR/HLA class I ligand combinations, and HLA class I in the development of CD and T1DM cooccurrence	KIR genes 2DS5 and 3DS1 were more frequent in patients with both diseases. The presence of HLA-C1 ligand was more frequent in the group with coexistence of CD and T1DM. Activating combinations of KIR genes and HLA-ligands were more frequent in the group with both diseases	The presence of KIR genes 2DS5, 3DS1, and HLA-C1 ligand are a risk factor for CD and coexistence of CD and T1DM, as well, as the activating combinations of genes and ligands

As seen in Table 2, all the reviewed studies showed an association between T1DM and CD, a fact we knew from previous research. But the new discoveries in pathogenesis and genetics are helping us to answer the previously unknown facts. We knew that HLA-DQ2.5 and DQ8 were the higher risk genes for developing both diseases, but now we know that the alleles DQA1*05 and DQB1*02 code for HLA-DQ2.5 and DQA1*03 and DQB1*03 code for HLA-DQ8 [14]. And thanks to the identification of the exact location of these alleles, we can use the HLA testing in high-risk patients to diagnose coexistence in asymptomatic patients [3]. However, due to the high percentage of positive alleles in T1DM patients, it is not recommended to test all patients because it is not cost-effective [16].

Akar et al. demonstrated a positive correlation between activating killer immunoglobulin-like receptor (KIR) genes, HLA class I ligand, and KIR/HLA class I ligand combinations, in patients with CD alone and in patients with T1DM and CD. Activating and inhibitory KIR genes control the activity of natural killer (NK) cells, which play a role in the pathogenesis of CD [16]. Their results are similar to other studies of the same class that show the impact of KIR genes in the development of CD [18]. This finding can be important in the elaboration of new strategies for the treatment of CD, especially in patients with baseline T1DM.

Microbiota

One of the newest advances in understanding the coexistence of CD and T1DM is the dysbiosis existing in patients with both diseases. The term "dysbiosis" is used to refer to an imbalance in normal gut microbiota which may cause different symptoms [19]. Patients with the coexistence of both diseases have similar bacterial gut composition, comparing them to patients with T1DM only, which can be attributed to the fact that patients develop T1DM before CD [20]. Also, the high levels of certain bacteria like *Bacteroides* and low levels of others like *Coprococcus* and *Prevotella* may play a role in the development of the classic symptomatology of CD, given that some bacteria use gluten as a fuel and the products of this metabolism may be the cause of the classic symptoms [7, 20, 21]. However, more studies are needed to clarify these statements. Table 3 contains the evidence found about the changes in microbiota in patients with the illnesses in study.

**Table 3:** Microbiota in patients with celiac disease and type 1 diabetes mellitus coexistence CD: celiac disease; HLA: human leukocyte antigen; T1DM: type 1 diabetes mellitus

Study	Author	Year	Type of study	Purpose of the study	Results	Conclusion
1	Singh et al. [20]	2021	Case-control	Evaluate gut microbiota in patients with CD and T1DM	CD+T1DM group had a different microbiome than healthy controls and similar to T1DM alone. High levels of *Shigella*, *Escherichia coli*, and especially *Bacteroides* were reported, and low levels of *Coprococcus*, *Prevotella*, *Alistipes*, *Oscillospira*, *Clostridium*, and microbiome diversity were reported in general	The gut microbiome is significantly different in patients with T1DM and T1DM+CD compared with healthy patients, which may explain the earlier diagnosis of CD in patients with T1DM
2	Cohn et al. [7]	2014	Review	Review genetic factors and gut microbiota contributing to CD and T1DM	Shared high-risk loci of HLA-DQ2 and DQ8 increased *Bacteroides* spp. and decreased lactate-producing and butyrate-producing bacteria	The gut microbiome seems to play a role in the development of CD and T1DM

Immune profile and environmental factors

Another discovery in pathogenesis is that patients with CD and T1DM have altered levels of cytokines, metalloproteinases, chemokines, acute phase proteins, and antibodies, compared with healthy controls. Tiberti et al. showed that patients with a previous diagnosis of T1DM had a low immune response at the time of diagnosis of CD years later. This finding may explain the difference in diagnosis time among different patients [22]. On the other hand, Tompa et al. found decreased levels of certain substances like interleukin-22 (IL-22), macrophage inhibitor potentiator protein (MIP-1α), monocyte chemoattractant protein-1 (MCP-1), acute phase proteins, procalcitonin, fibrinogen, adipocytokine visfatin, and matrix metalloproteinases (MMP-2). This finding may confer a protective role in the development of CD in patients with T1DM [8]. Another research like Guan et al. studied the levels of specific cytokines like MCP-1 and showed low levels of it in patients with T1DM compared with healthy controls [23]. Similarly, El Samahi et al. studied the levels of intracellular enzyme visfatin in patients with T1DM, showing lower levels compared with healthy controls [24]. All of this information demonstrates that the low levels of cytokine, enzyme, proteins, etc. may play a protective role in the pathogenesis of T1DM and CD. However, as suggested in the papers previously reviewed, studies with greater samples are needed to have better conclusions.

Opposite to the previous discussion, Vorobjova et al. found increased levels of certain cytokines such as IL-17F, interferon-gamma-induced protein (IP-10), soluble tumor factor receptor 2 (sTNFRII), MCP-1, and granulocyte-macrophage colony-stimulating factor (GM-CSF), which correlates with the level of inflammation and enterovirus infection in the gut cells of patients with CD and T1DM [25]. This finding is also present in the study of Goodwin, who, in his review, also said that the presence of enterovirus in gut cells had increased the risk and was present in patients with coexistence of T1DM and CD [2]. This can be corroborated with the results of the TEDDY study (The Environmental Determinants of Diabetes in the Young) which was conducted with a sample of 8676 children, where they strongly demonstrated that the enterovirus infection predisposes to the development of CD in diabetic patients [26].

Another fact to mention is that Åkesson et al. had shown an elevated percentage of terminally differentiated helper T cells CD4+ and a decreased amount of effector memory cytotoxic T cells, early differentiated and late differentiated CD8+ [9]. However, the authors concluded that their findings were not conclusive due to the differences with other studies in which the levels of CD4+ and CD8+ cells were positively correlated with autoimmunity and reactivity to gluten in CD [27]. In Table 4, we summarize the findings of the studies previously mentioned.

**Table 4:** Immune profile and environmental factors in patients with celiac disease and type 1 diabetes coexistence CD: celiac disease; GM-CSF: granulocyte-macrophage colony-stimulating factor; IL: interleukin; IP-10: interferon gamma-induced protein 10; MCP-1: monocyte chemoattractant protein 1; MIP-1α: macrophage inhibitor potentiator protein 1 alpha; MIP-1b: macrophage inhibitor potentiator protein 1 beta; sIL-2Rα: soluble interleukin 2 receptor alpha; sTNFRII: soluble tumor factor receptor II; T1DM: type 1 diabetes mellitus; EV: enterovirus

Study	Author	Year	Type of study	Purpose of the study	Results	Conclusion
1	Tompa et al. [8]	2020	Case-control	Study the peripheral blood immune profile in patients with T1DM and CD	Decreased levels of chemokines IL-22, MIP-1α, MCP-1; acute phase proteins procalcitonin and fibrinogen; adipocytokine visfatin; matrix metalloproteinases MMP-2	Low Visfatin, IL-22 levels may be a protective factor in the development of CD in patients with T1DM. Further research is needed to explain the variation in MMP levels between studies
2	Vorobjova et al. [25]	2019	Case-control	Compare cytokine profiles between patients with and without CD and T1DM. Evaluate if cytokine levels are related to EV infection	Patients with CD and T1DM had higher cytokine levels than controls. Levels of cytokines were positively correlated with IgG levels to EV	Increased levels of IL-15, IL-17F, MIP-1b, and sIL-2Rα in patients with CD and T1DM High levels of IL17F, IP-10, sTNFRII, MCP-1, and GM-CSF in EV positive cells
3	Goodwin. [2]	2019	Review	Analyze environmental and non-genetic factor associated with the development of CD and T1DM	Exposure to gluten before 4 months of life increases the risk of developing autoimmunity >5 respiratory infections per year exponentially increase the risk of T1DM EV infection of gut cells increase in 10-fold the risk of T1DM and CD	The combination of genetic predisposition and environmental factors definitely contribute to the autoimmune process of T1DM and CD
4	Åkesson et al. [9]	2015	Cohort	Characterize the CD4+ and CD8+ cell panel in patients with T1DM and/or CD	An elevated amount of terminally differentiated helper T cells in the CD4+ lymphocytes. Decreased amount of effector memory cytotoxic T cells, early differentiated and late differentiated CD8+	Patients with both diagnoses have a higher percentage of helper T cells CD4+ compared with cytotoxic T cells
5	Tiberti et al. [22]	2012	Cross-sectional	Compare the celiac-specific immune response between T1DM and non-diabetic CD patients at the time of diagnosis	Antibody titers were significantly less in patients with a previous diagnosis of T1DM compared with non-diabetics	The previous T1DM status strongly correlates with the low immune response at the time of CD diagnosis

Limitations

During this review, we found some limitations. First, the information obtained was from one database only (PubMed). Second, various types of studies were included, including cohorts, cross-sectionals, reviews, and case controls; therefore, the differences in samples and statistic methods used in the different studies make our study prone to bias. Third, the majority of papers included were done with small samples, and in different stages of the diseases. Thus, studies with greater samples are needed in order to get more conclusive results.

## Conclusions

We aimed to review the different pathogenic factors that are proposed to play a role in the coexistence of CD and T1DM, which is important to understand them and to potentially use this information for the improvement of new treatment strategies. To conclude, we can say that HLA-DQ2.5 and HLA-DQ8 are the higher risk genes to develop coexistence of these diseases, basically its presence tells us why these two appear together in a great amount of cases; also, the testing of these genes can be beneficial in the diagnosis of CD and T1DM depending on the clinical condition of every specific patient. Additionally, that KIR genes controlling NK cell activity have a crucial role in the development of clinical symptoms of CD in patients with previous T1DM diagnosis. One new discovery that is gaining terrain in this field is that dysbiosis in gut microbiota has also played a very important role in the development of the symptoms of CD. The reviewed studies emphasized the elevated presence of *Bacteroides* spp. and gluten-using bacteria. In the same way, the evidence shows a clear role of enterovirus infection in gut cells and degree of inflammation. Finally, we have seen an imbalance in the immune profile of patients with the coexistence of CD and T1DM, especially referring to the levels of CD4+ and CD8+ cells. All these factors can give us a brief overview of why these illnesses vary so widely among individuals. However, due to the discrepancy between studies on these topics, we suggest additional research with greater samples and statistically similar control groups to obtain definitive results.
